# Measuring the relationship between outpatient family physician visit regularity and acute care utilization during the end of life: a population-level retrospective cohort study

**DOI:** 10.1093/fampra/cmag014

**Published:** 2026-04-07

**Authors:** Shuaib Hafid, Aaron Jones, Anastasia Gayowsky, Aria Wills, Sarina R Isenberg, Michelle Howard

**Affiliations:** Department of Family Medicine, McMaster University, Hamilton, Ontario, Canada L8P 1H6; Department of Health Research Methods, Evidence and Impact, McMaster University, Hamilton, Ontario, Canada L8S 4L8; Department of Health Research Methods, Evidence and Impact, McMaster University, Hamilton, Ontario, Canada L8S 4L8; ICES McMaster University, Hamilton, Ontario, Canada L8S 4K1; ICES McMaster University, Hamilton, Ontario, Canada L8S 4K1; Bruyere Health Research Institute, Ottawa, Ontario, Canada K1N 5C8; Bruyere Health Research Institute, Ottawa, Ontario, Canada K1N 5C8; Department of Medicine, University of Ottawa, Ottawa, Ontario, Canada K1H 8M5; Department of Family Medicine, McMaster University, Hamilton, Ontario, Canada L8P 1H6

**Keywords:** family physician, continuity of care, regularity, end-of-life care, cardiorespiratory

## Abstract

**Background:**

Family physicians (FP) are critical in providing outpatient end-of-life (EOL) care, yet most continuity measures do not consider temporal changes in visits. The relative variance index (RVI), which measures visit interval variation, has not been applied to the EOL context.

**Aim:**

To measure the association between outpatient FP visit regularity & acute care use in the last month of life.

**Design:**

Retrospective cohort study using linked population-level health administrative data. FP visit regularity was measured using the RVI, calculated from outpatient visits during the last two years of life (excluding the last month). Outcomes included hospitalizations, emergency department (ED) visits in the last month of life & acute care setting deaths.

**Setting:**

Adults with cardiorespiratory conditions who died in Ontario, Canada, between 2017 & 2019.

**Results:**

Patients' (*N* = 151 030) median age at 2-years before death was 80, 55% were male, & had a median of 9 FP outpatient visits in the last two years of life. Higher FP RVI scores were associated with increased hospitalizations (IRR [95% CI]: 1.07 [1.05,1.10]), ED visits (1.06 [1.03, 1.08]) & acute care deaths (OR [95% CI]: 1.30 [1.26, 1.35]). Sensitivity analyses identified that RVI scores varied across different look-back periods, while sensitivity model performances remained stable when adjusting for specialist visit regularity.

**Conclusions:**

Higher outpatient FP visit regularity was associated with increased EOL acute care use, despite low visit regularity during the last two years of life. However, the RVI's sensitivity to observation period lengths limits its utility as an EOL care-quality indicator.

Key messages

- Family doctors play a key role in end-of-life care, but visit regularity is generally low.
- More regular community (i.e. office, home, or phone) visits with family doctors were linked to slightly higher hospital and ER use, but other patient factors had a stronger impact.
- Patient complexity and home care access influenced end-of-life outcomes more than visit regularity.
- The relative variance index is sensitive to timing and exposure period definitions, limiting its use as a care quality measure.
- Better tools are needed to measure continuity of care in end-of-life settings.

## Introduction

Family physicians are essential in the Canadian healthcare system—acting as both gatekeepers to specialist care and coordinators for medically complex patients [1–3]. This role is especially important during the end-of-life phase; for example, family physicians are responsible for most outpatient visits during the last year of life among people who died in Ontario, Canada [4]. This dynamic is also observed in the United Kingdom and other similar countries [5, 6].

Continuity of care, defined as the coherence and connectedness of healthcare events with patient needs, is a quality indicator linked to reduced adverse outcomes and acute care use [7–11]. Continuity indices have typically measured the concentration of care within specific physicians [12] or the dispersion of care across physicians [13]. However, these indices do not make temporal considerations, such as the regularity of visits with physicians [14]. Patients may experience identical continuity despite having completely different temporal concentrations of care (i.e. the points in time where visits occur relative to the patients' death date). As a result, considering the temporal patterns in visits may influence the degree of management and longitudinal, or relational, continuity patients experience with their family physicians. For instance, patients who regularly visit their family physician may experience improved care outcomes as their family physician may be actively managing their care, resulting in proactive discussions regarding goals of care.

The relative variance index (RVI) aims to address this pitfall by measuring the variability in interval durations (i.e. in days) between consecutive physician visits [14]. The index was developed for the purpose of decoupling visit regularity from the overall care frequency of family physician visits. However, the index has not been applied at the end of life. Understanding whether outpatient family physician visit regularity affects end-of-life healthcare outcomes is important as Canada's aging population and family physician shortages intensify the need for effective end-of-life care [15–22]. Theoretically, patients with higher physician visit regularity should experience improved longitudinal continuity and, therefore, may experience improved end-of-life outcomes. This study measures the association between patients' outpatient family physician visit regularity in the last two years of life and patients' acute care utilization in the last 30 days of life and their location of death.

## Methods

### Study design & data sources

We conducted a retrospective cohort study using linked population-level health administrative data from Ontario, Canada (see Supplementary Appendix S1 for a description of the data holdings accessed). Administrative data holdings are linked using unique encoded identifiers and analyzed at ICES (formerly known as the Institute for Clinical Evaluative Sciences). ICES is an independent, nonprofit research institute whose legal status under Ontario's health information privacy law allows it to collect and analyze healthcare and demographic data for health system evaluation and improvement. This study received ethics approval from the Hamilton Integrated Research Ethics Board (28 March 2022; #14750-C).

### Study population

**Figure 1:**
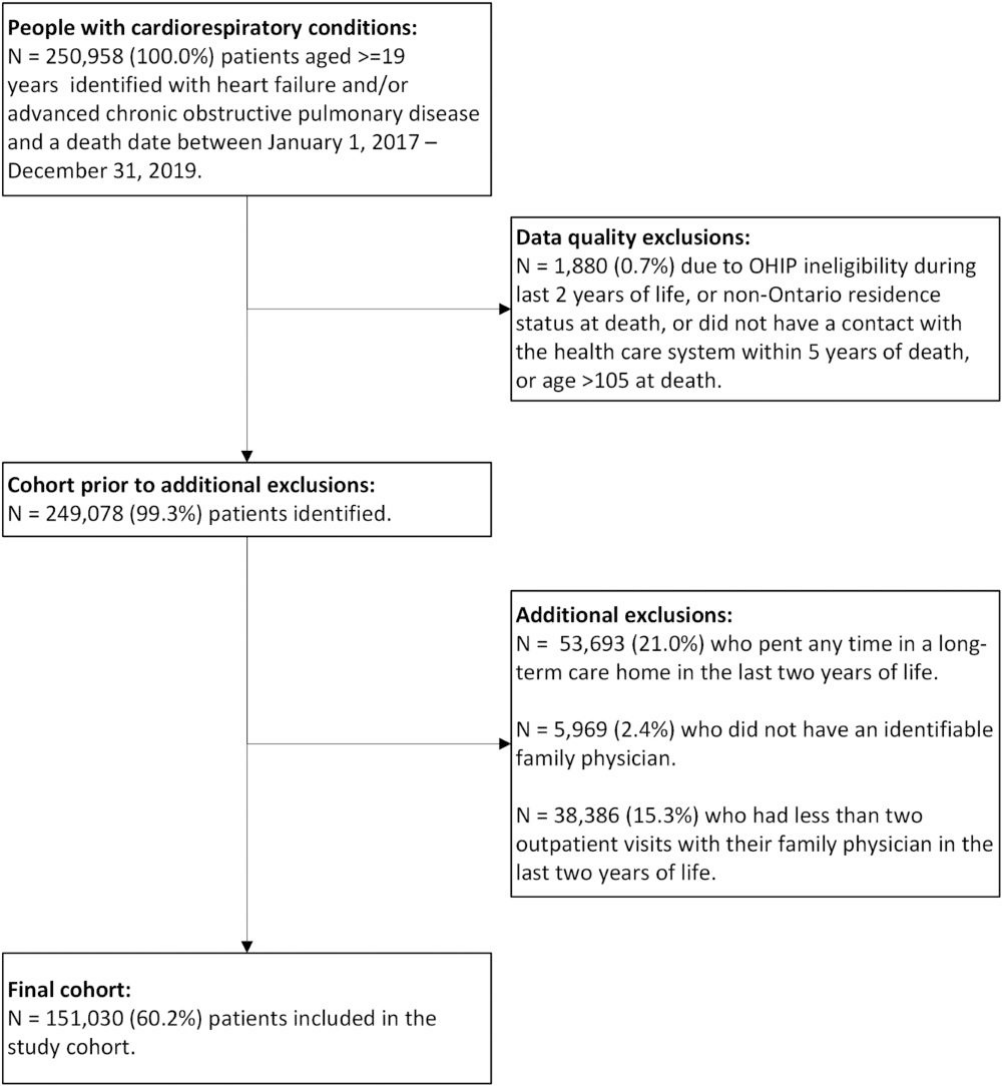
Cohort creation flowchart.

We included adults (aged 19 or older) who died between 1 January 2017, and 31 December 2019, with cardiorespiratory conditions—specifically advanced chronic obstructive pulmonary disease (ACOPD) and/or heart failure (HF). We focused on this patient population as these individuals are medically complex (i.e. have multiple comorbidities) [23, 24], have less predictable illness trajectories, and often are not deemed as “palliative” [16, 25], resulting in being managed primarily by family physicians at the end of life [23, 24]. Using a validated COPD case definition [26], we identified COPD prevalence 2 years before death and identified ACOPD as having at least one hospitalization with a COPD-related diagnosis and/or receipt of long-term oxygen therapy in that period. For HF, we adapted a validated case definition [27] that required any inpatient or outpatient visit with an HF diagnosis, followed by a confirmatory record within a year—without restricting to those ≥65 years as our cohort consists of decedents. Patients were excluded from the cohort if they were 105 years of age or older at death to exclude patients with potential administrative errors in their dates of birth or death (Fig. 1), and if they were ineligible for the Ontario Health Insurance Plan (OHIP) at any point in their last two years of life. Patients were also excluded if they resided in a long-term care home during the last two years of life as long-term care home residents may receive different patterns of outpatient care compared to community-dwelling patients. Lastly, we excluded patients with fewer than two outpatient family physician visits during the last two years of life to ensure that the exposure variable could be calculated.

### Exposures

The study exposure was outpatient visit regularity with their rostered family physician, which was calculated using the RVI (Supplementary Appendix S2 for the formula). The RVI, ranging from 0 (irregular) to 1 (perfectly regular), was calculated from visits occurring during the two years before death, truncating the final 30 days. To assess the effects of increasing outpatient family physician visit regularity, the RVI scores were categorized into empirical quintiles. We also calculated the RVI scores of outpatient visits with all specialists (treated collectively, instead of individually) for two reasons: (1) to understand the correlation between family physician visit regularity and specialist visit regularity; and (2) to conduct sensitivity models including outpatient specialist visit regularity. Outpatient visits were defined as unique OHIP physician billing claims where the location code was “Office”, “Home”, or “Phone”. Patients' family physicians were identified using the Client Agency Program Enrolment table. If patients were not formally attached to a family physician, they were virtually attached to the family physician with the highest primary care billings in the two years before the last two years of life [28]. Specialist outpatient encounters were limited to specialties for which patients receive direct non-family physician care from (i.e. excluding emergency medicine, pathology, etc.).

### Outcomes

The study outcomes include the following: (1) new hospitalizations during the last 30 days of life; (2) emergency department (ED) visits in the last 30 days of life; and (3) whether death occurred in an acute care setting. These outcomes are commonly measured as end-of-life care quality indicators [29, 30], and are considered to occur at higher rates when there is limited access to quality end-of-life care in the community at the system-level. Unique hospitalizations and ED visits were captured using the Discharge Abstract Database (DAD) and the National Ambulatory Care Reporting System (NACRS), respectively. Acute care deaths were captured using an ICES-developed SAS macro, which leverages the DAD, NACRS, and other administrative data sources.

### Covariates

Covariates were selected *a priori* and measured at 2 years before death (unless otherwise specified), including: (1) age; (2) sex; (3) rurality status; (4) neighborhood income quintile status; (5) comorbidity status; (6) the number of outpatient visits with their family physician during the last two years of life (truncating the last 30 days); and (7) the number of visits to all other specialists during the last two years of life (truncating the last 30 days). Demographic covariates were defined using the Registered Persons Database (i.e. age and sex) and Statistics Canada's Postal Code Conversion File+, linked to the 2016 Census Data (i.e. rurality status and neighborhood income quintiles). Comorbidity status was captured using the Johns Hopkins Adjusted Clinical Group (ACG)® System Aggregated Diagnosis Groups (ADGs), Version 10 [31, 32], to report comorbidity status at one year before death. The ACG system categorizes all assigned ICD-9/ICD-9-CM diagnosis codes into 32 functional groups, based on the certainty of diagnosis, duration, severity, and etiology of the condition, as well as the odds of needing specialist services. Therefore, we applied the ACG system across all available health administrative data sources to identify the number of prevalent ADGs 2 years before death for each patient.

### Analyses

Descriptive statistics are presented with medians and (25th percentiles, 75th percentiles) for continuous variables and counts (percentages) for categorical variables. Associations between RVI quintiles and count-based outcomes were analyzed with negative binomial regressions (log-link), while logistic regression (logit-link) was used for the dichotomous outcome of acute care death. Models were adjusted for both exposures and covariates, and incidence rate ratios (IRR) and odds ratios (OR) with 95% confidence intervals (CI) were reported. Statistical significance was set at *P*-value <.05 (two-tailed) and analyses were completed using R Studio V.0.98.1091.

### Correlation & sensitivity analysis

Pearson correlation coefficients were calculated among outpatient family physician visit RVI scores, outpatient specialist visit RVI scores, and outpatient utilization measures such as the number of family physician visits, the number of specialist visits, and the number of unique specialists visited during the last two years of life (truncating the last 30 days of life). This analysis aimed to determine whether the RVI adds unique predictive value for end-of-life healthcare outcomes beyond overall visit frequency, as their shared variance would be higher. Additionally, sensitivity analyses assessed the index's stability by using different observation periods: (1) two years to three months before death; (2) two years to one year before death; and (3) one year to three months before death. Lastly, we conducted a sensitivity model adjusting for the outpatient specialist visit RVI scores to control for effects of specialist visit regularity on the family physician regularity during the last two years of life, and we produced Akaike Information Criterion (AIC) scores to compare model goodness-of-fit performances.

## Results

After exclusions, 151 030 eligible individuals who died with cardiorespiratory conditions were analyzed (Fig. 1). At two years before death, median (P25, P75) age was 80 (71, 87), 55.3% were male, 87.3% lived in urban regions, and 47.8% resided in lower-income neighborhoods (Table 1). The median number of ADGs was 13 (11, 16), 35.1% had cancer at, and 76.4% received provincial homecare services during the last two years of life. 18.2% of patients had both ACOPD and HF 2 years before death, 13.0% had ACOPD only, and 68.8% had HF only. Patients with ACOPD alone were younger than those with both ACOPD and HF or with HF alone, had the highest cancer prevalence at two years prior to death, and had a slightly lower proportion of males. In contrast, patients with both ACOPD and HF were the most complex, with the highest number of ADGs and the greatest enrollment in provincial homecare services. All characteristics were significantly different across the cardiorespiratory condition subgroups (*P* < .001).

**Table 1:** Profile of people aged 19 or older with ACOPD and/or HF who died between 1 January 2017, and 31 December 2019, in Ontario, Canada.

Variable	Level	Total cohort N = 151 030	ACOPD & HF N = 27 512 (18.2%)	ACOPD only N = 19 641 (13.0%)	HF only N = 103 877 (68.8%)	*P*
Age	Median (P25, P75)	80 (71, 87)	79 (71, 86)	74 (66, 81)	81 (72, 87)	<.001
Sex	Male	83 486 (55.3%)	14 937 (54.3%)	10 142 (51.6%)	58 407 (56.2%)	<.001
Nearest census-based neighborhood income quintile	Q1—lowest	37 869 (25.1%)	7453 (27.1%)	5363 (27.4%)	25 053 (24.2%)	<.001
	Q2	34 232 (22.7%)	6271 (22.8%)	4448 (22.7%)	23 513 (22.7%)	
	Q3	29 177 (19.4%)	5194 (18.9%)	3758 (19.2%)	20 225 (19.5%)	
	Q4	25 401 (16.9%)	4563 (16.6%)	3184 (16.3%)	17 654 (17.0%)	
	Q5—highest	23 911 (15.9%)	3971 (14.5%)	2824 (14.4%)	17 116 (16.5%)	
Rurality status	Yes	19 093 (12.7%)	4177 (15.2%)	3290 (16.8%)	11 626 (11.2%)	<.001
Received provincial homecare services during the last 2 years of life	Yes	115 315 (76.4%)	23 867 (86.8%)	15 687 (79.9%)	75 761 (72.9%)	<.001
Cancer prevalence	Yes	52 981 (35.1%)	9806 (35.6%)	7405 (37.7%)	35 770 (34.4%)	<.001
Number of prevalent Johns Hopkins adjusted diagnostic groups	Median (P25, P75)	13 (11, 16)	14 (12, 16)	13 (10, 15)	13 (11, 16)	<.001

ACOPD, advanced chronic obstructive pulmonary disease; HF, heart failure; P25, 25th percentile; P75, 75th percentile.

### Healthcare utilization & RVI scores

During the last two years of life (excluding the last 30 days of life), individuals had a median of 9 (5, 15) visits with their family physician and 12 (6, 21) visits with specialists; with a median of 5 (3, 8) unique specialist physicians involved (Table 2). Patients with ACOPD and HF had the highest number of visits, compared to patients with either ACOPD or HF only (*P* < .001). Patients had a median RVI score of 0.011 (0.008, 0.014) with their FP and a score of 0.009 (0.007, 0.011) with their specialists; differences across cardiorespiratory condition subgroups were statistically—but not clinically—significant. In the last 30 days of life, the median number of new hospitalizations was 1 (0, 1) and 1 (0, 1) for ED visits (Table 3). However, the percentages of acute care deaths varied across the cardiorespiratory condition subgroups: 59.9% for individuals with ACOPD and HF, 48.0% for ACOPD only, and 64.8% for HF only.

**Table 2:** Regularity of outpatient visits among people with ACOPD and/or HF between 1 January 2017, and 31 December 2019, in Ontario, Canada.

Variable	Total cohort N = 151 030	ACOPD & HF N = 27 512 (18.2%)	ACOPD only N = 19 641 (13.0%)	HF only N = 103 877 (68.8%)	*P*
Family physician visits, Median (P25, P75)	9 (5, 15)	10 (6, 17)	9 (5, 14)	9 (5, 15)	<.001
Specialist visits, Median (P25, P75)	12 (6, 21)	13 (6, 22)	12 (6, 21)	11 (5, 20)	<.001
Unique specialists, Median (P25, P75)	5 (3, 8)	5 (3, 8)	4 (3, 7)	5 (3, 8)	<.001
Family Physician RVI, Median (P25, P75)	0.011 (0.008, 0.014)	0.011 (0.008, 0.014)	0.012 (0.008, 0.014)	0.012 (0.008, 0.014)	<.001
Specialist RVI, Median (P25, P75)	0.009 (0.007, 0.011)	0.009 (0.008, 0.011)	0.009 (0.007, 0.011)	0.009 (0.007, 0.012)	<.001

ACOPD, advanced chronic obstructive pulmonary disease; HF, heart failure; P25, 25th percentile; P75, 75th percentile; RVI, relative variance index.

**Table 3:** Acute care utilization during the last month of life among people with ACOPD and/or HF between 1 January 2017, and 31 December 2019, in Ontario, Canada.

Variable	Total cohort N = 151 030	ACOPD & HF N = 27 512 (18.2%)	ACOPD only N = 19 641 (13.0%)	HF only N = 103 877 (68.8%)	*P*
Number of new hospital admissions in the last 30 days of life, Median (P25, P75)	1 (0, 1)	1 (0, 1)	1 (0, 1)	1 (0, 1)	<.001
Number of new emergency department visits in last 30 days of life, Median (P25, P75)	1 (0, 1)	1 (0, 1)	1 (0, 1)	1 (0, 1)	<.001
Acute care death, n (%)	93 215 (61.7%)	16 488 (59.9%)	9426 (48.0%)	67 301 (64.8%)	<.001

ACOPD, advanced chronic obstructive pulmonary disease; HF, heart failure; P25, 25th percentile; P75, 75th percentile.

### Associations with acute care use at the end of life

**Figure 2:**
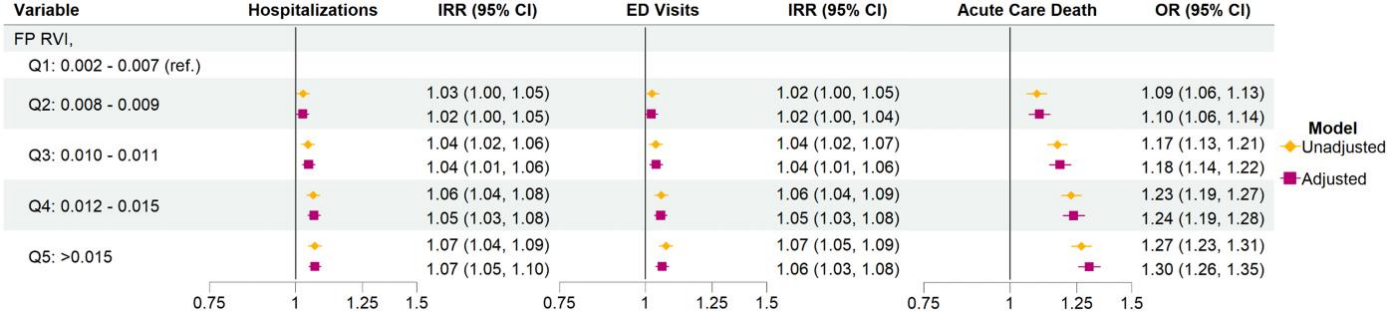
Associations between outpatient family physician visit relative variance index scores and acute care utilization during the last 30 days of life.

The highest outpatient family physician visit RVI quintile was associated with increased new hospitalizations (IRR 1.07 [1.05, 1.10]), ED visits (IRR 1.06 [1.03, 1.08]), and odds of dying in an acute care hospital (OR 1.30 [1.26, 1.35]) compared to the lowest quintile (Fig. 2). Notably, covariates such as the receipt of homecare services, cancer prevalence, and higher comorbidity status demonstrated much larger effect sizes on the EOL outcomes compared to the outpatient family visit RVI scores (Supplementary Appendix S3). For example, patients receiving provincial homecare services were less likely to use acute care at the EOL (hospitalizations: 0.96 (0.94, 0.97); ED visits: 0.50 (0.49, 0.52); and acute care deaths (0.83 (0.82, 0.84) after adjustment. Similarly, cancer prevalence was also associated with decreased use, while increasing comorbidity status was associated with increases in acute care use.

### Correlation & sensitivity analysis results

Correlation analyses identified that FP and specialist RVI scores were largely independent of the overall number of outpatient visits or the number of physicians involved during the EOL (Supplementary Appendix S5). These results confirm that the index captures different dimensions of healthcare utilization, aligning with its initial conceptualization [14]. However, the sensitivity analyses revealed that RVI scores vary considerably with different definitions for the EOL period (Supplementary Appendix S6). For instance, the median FP RVI decreased to 0.009 when excluding the last 90 days of life, highlighting the index's sensitivity to timing. Lastly, associations between outpatient family physician visit regularity and end-of-life acute care use persisted after adjusting for outpatient specialist visit regularity, with Akaike Information Criterion scores only marginally decreasing with inclusion (Supplementary Appendix S3 and S4).

## Discussion

This population-level study of patients who died with cardiorespiratory conditions identified that outpatient family physician visit regularity is generally low during the last two years of life Counterintuitively, higher family physician regularity—as measured by the RVI—was still associated with modest increases in acute care use during the last 30 days of life (increased rates of hospitalizations and ED visits, and odds of dying in an acute care setting). However, the effects of patients' medical complexity (e.g. higher comorbidity status and cancer prevalence) overshadowed the associations between outpatient family physician visit RVI scores and acute care use during the end of life. This finding is unsurprising as medically complex patients will naturally have more specialists involved, and as a result, may inevitably require more acute care during the end of life regardless of their prior outpatient family physician visit regularity. The belief that higher visit regularity confers better coordination and improved end-of-life outcomes may not be valid when patient complexity predominates their care provision. These findings align with previous research suggesting that while continuity improves outcomes [7], other factors (such as comorbidity burden) play a more significant role in determining end-of-life care trajectories. Meanwhile, patients' who received provincial homecare services were associated with large decreases in acute care utilization. This finding is also expected as enrolment results in the involvement of palliative care providers, including physicians and nurse practitioners. Therefore, homecare recipients may receive a palliative approach to care, which may inform their goals of care, resulting in decreased care use. This complements existing literature that identified that homecare recipients were less likely to die in hospitals [33]. As a result, it is difficult to confidently ascertain the effects of increased visit regularity on acute care use during the end of life when other associations are substantially stronger. Ultimately, the RVI may not be an appropriate predictor of end-of-life care quality due to these clinical limitations.

Methodologically, the RVI is a promising indicator as it considers the timing of visits instead of solely focusing on visit concentration or dispersion. However, there are a few methodological concerns. First, the raw RVI scores calculated were very low but consistent with previous applications among older adults (although these studies did not measure associations with end-of-life care outcomes). Therefore, the index must be dichotomized into empirical quantiles to be meaningfully interpretable [14, 34, 35]. While this approach improves internal comparability, it hampers external validity. The reported rank order of the quantiles is relative to the patient population at hand, and as a result, different patient populations with varying interval distributions will be incomparable. Compounded by using any preferred quantile unit (i.e. terciles, quartiles, quintiles, deciles), the relative nature of the index limits its utility as a system-level predictor of care quality across different populations. Second, it is highly improbable that patients will experience perfect outpatient family physician visit regularity due to the inherent unpredictability present at the end of life. This concern is further complicated by the index's sensitivity to minor deviations in interval durations. As a result, two patients with virtually identical interval patterns can have substantially different RVI scores. For example, patient A, with five intervals that are each 30 days apart, results in a score of 1.0 (perfect regularity), while patient B, who also had five intervals, where the first four are 30 days apart, and the last interval is 31 days apart, results in an RVI score of 0.40. This raisesthe question of the index's precision in measuring regular visitation, notwithstanding that patients are more likely to experience highly irregular intervals compared to the hypothetical example presented due to the number of different physicians involved during the end of life. Lastly, our sensitivity analyses demonstrate that the RVI is highly dependent on the chosen exposure period. For instance, excluding the last 30 days versus the last 90 days of life produced different scores. This variability is challenging for EOL research since exposure periods are typically anchored to the individuals' death dates, yet outpatient visits often cluster near death [4]. Moreover, setting the exposure start date relative to death is arbitrarily selected (i.e. one year versus two years) and may exclude encounters occurring just outside the defined interval.

### Strengths & limitations

This study has several strengths. First, we used routinely collected population-level health administrative data, which minimizes the degree of missingness while maximizing external validity. Therefore, the study findings may be relevant to patients dying with cardiorespiratory conditions in other comparable jurisdictions. Moreover, the large sample size and linkages to other datasets allowed for detailed adjustment of relevant covariates. Lastly, this study represents a novel application of the RVI as a care quality predictor for the end of life.

This study also has limitations. First, the cohort was limited to individuals who were attached to a family physician or individuals with at least two outpatient visits with their usual family physician during the exposure period. Therefore, our findings may not generalize to patients with less consistent primary care access. Next, we did not consider the potential group effects due to clustering at the physician-level. Patients with the same family physicians may experience similar visit regularity due to their physician's practice characteristics. Moreover, patients may receive primary care services from non-physicians (i.e. nurse practitioners) in Ontario, Canada. However, this data is not captured in our provincial health administrative holdings. Lastly, cause of death information was unavailable for our study cohort at the time of study completion; therefore, our cohort included individuals who died *with* cardiorespiratory conditions and not *of* these conditions. As a result, we cannot assess the effects of their cause of death on their end-of-life care in relation to outpatient visit regularity.

## Conclusions

Among patients who died with cardiorespiratory conditions, outpatient visit regularity with family physicians was very irregular during the end of life. Although higher regularity is associated with increased acute care use—including more hospitalizations, ED visits, and higher odds of acute care deaths—the observed effect sizes are substantially lower than those associated with patient complexity and homecare service use. Moreover, the RVI's sensitivity to the defined exposure period and minor interval variations challenges it utility as a stand-alone care quality predictor for end-of-life care. Future research should qualitatively explore patient perceptions of outpatient family physician visit regularity at the end of life to better inform new temporal continuity measures that reflect the unique and dynamic nature of end-of-life care.

## Supplementary Material

cmag014_Supplementary_Data

## Data Availability

The data set from this study is held securely in coded form at ICES. While data sharing agreements prohibit ICES from making the data set publicly available, access may be granted to those who meet pre­specified criteria for confidential access, available at www.ices.on.ca/DAS. The full data set creation plan and underlying analytic code are available from the authors upon request, understanding that the computer programs may rely upon coding templates or macros that are unique to ICES and are therefore either inaccessible or may require modification.

## Prior presentations

2025 McMaster HEI Research Day, 29 April 2024, Burlington, Canada.

## References

[1] Easley J, Miedema B, Carroll JC et al Coordination of cancer care between family physicians and cancer specialists: importance of communication. Can Fam Physician 2016;62:e608–15. 27737996 PMC5063787

[2] Easley J, Miedema B, O’Brien MA et al The role of family physicians in cancer care: perspectives of primary and specialty care providers. Current Oncology 2017;24:75–80. 10.3747/co.24.3447 28490920 PMC5407869

[3] Chaput G, Sussman J. Integrating primary care providers through the seasons of survivorship. Current Oncology 2019;26:48–54. 10.3747/co.26.4687 30853798 PMC6380650

[4] Howard M, Hafid A, Isenberg SR et al Intensity of outpatient physician care in the last year of life: a population-based retrospective descriptive study. CMAJ Open 2021;9:E613–22. 10.9778/cmajo.20210039 PMC8191591 34088732

[5] Mitchell S, Loew J, Millington-Sanders C et al Providing end-of-life care in general practice: findings of a national GP questionnaire survey. British Journal of General Practice 2016;66:e647–53. 10.3399/bjgp16X686113 PMC5198709 27381483

[6] Mitchell S, Oliver P, Gardiner C et al Community end-of-life care during the COVID-19 pandemic: findings of a UK primary care survey. BJGP Open 2021;5:BJGPO.2021.0095. 10.3399/bjgpo.2021.0095 34117014 PMC8450890

[7] Haggerty JL, Reid RJ, Freeman GK et al Continuity of care: a multidisciplinary review. BMJ: British Medical Journal 2003;327:1219–21. 10.1136/bmj.327.7425.1219 14630762 PMC274066

[8] Brener SS, Bronksill SE, Comrie R et al Association between in-hospital supportive visits by primary care physicians and patient outcomes: a population-based cohort study. J Hosp Med 2016;11:418–24. 10.1002/jhm.2561 26914153

[9] Morey T, Scott M, Saunders S et al Transitioning from hospital to palliative care at home: patient and caregiver perceptions of continuity of care. J Pain Symptom Manage 2021;62:233–41. 10.1016/j.jpainsymman.2020.12.019 33385479

[10] Cabana MD, Jee SH. Does continuity of care improve patient outcomes? J Fam Pract 2004;53:974–80. 15581440

[11] Saultz JW . Defining and measuring interpersonal continuity of care. Ann Fam Med 2003;1:134–43. 10.1370/afm.23 15043374 PMC1466595

[12] Reid R, Haggerty J, McKendry R. Defusing the confusion: concepts and measures of continuity of health care. Ottawa, ON: Canadian Health Services Research Foundation, 2002.

[13] Bice TW, Boxerman SB. A quantitative measure of continuity of care. Med Care 1977;15:347–9. 10.1097/00005650-197704000-00010 859364

[14] Youens D, Harris M, Robinson S et al Regularity of contact with GPs: measurement approaches to improve valid associations with hospitalization. Fam Pract 2019;36:650–6. 10.1093/fampra/cmz002 30689822

[15] Lunney JR, Lynn J, Foley DJ et al Patteerns of functional decline at the End of life. J Am Med Assoc 2003;289:2387–92. 10.1001/jama.289.18.2387 12746362

[16] Fassbender K, Fainsinger RL, Carson M, et al Cost trajectories at the End of life: the Canadian experience. J Pain Symptom Manage 2009;38:75–80. 10.1016/j.jpainsymman.2009.04.007 19615630

[17] Mathers CD, Loncar D. Projections of global mortality and burden of disease from 2002 to 2030. PLoS Med 2006;3:e442. 10.1371/journal.pmed.0030442 17132052 PMC1664601

[18] Rosella L, Kornas K, Huang A et al Accumulation of chronic conditions at the time of death increased in Ontario from 1994 to 2013. Health Aff 2018;37:464–72. 10.1377/hlthaff.2017.1150 29505380

[19] Statistics Canada . *Census in Brief, A portrait of Canada's growing population* aged 85 and older from the 2021 Census, Census of Population, 2021. https://www12.statcan.gc.ca/census-recensement/2021/as-sa/98-200-X/2021004/98-200-X2021004-eng.pdf (08 September 2023, date last accessed).

[20] Kiran T, Green ME, Wu CF et al Family physicians stopping practice during the COVID-19 pandemic in Ontario, Canada. Ann Fam Med 2022;20:460–3. 10.1370/afm.2865 36228068 PMC9512549

[21] Abdulla A. *Abdulla: You want a family doctor in Ontario? Sorry, it's not going to be easy.* Ottawa Citizen*. 2023–05–03*. https://ottawacitizen.com/opinion/abdulla-you-want-a-family-doctor-in-ontario-sorry-its-not-going-to-be-easy (08 September 2023, date last accessed).

[22] Board SE. *An unhealthy shortage of family doctors. Toronto Star*. https://www.thestar.com/opinion/editorials/an-unhealthy-shortage-of-family-doctors/article_6b3e3e48-ff56-5e32-b2fd-f464f4febcc3.html (08 September 2023, date last accessed).

[23] Fernandes A, Shuaib H, Anastasia G et al Care utilization patterns among patients dying with advanced chronic obstructive pulmonary disease. Can J Respir Crit Care Sleep Med 2025;9:1–9. 10.1080/24745332.2024.2437412

[24] Hafid S, Wills A, Quinn KL et al Patterns of healthcare delivery among adults with heart failure in the last year of life: a retrospective population-based study. J Am Heart Assoc 2025;14:e038189. 10.1161/JAHA.124.038189 40365778 PMC12184604

[25] Murray SA, Kendall M, Boyd K et al Illness trajectories and palliative care. BMJ 2005;330:1007–11. 10.1136/bmj.330.7498.1007 15860828 PMC557152

[26] Gershon AS, Maclagan LC, Luo J et al End-of-life strategies among patients with advanced chronic obstructive pulmonary disease. Am J Respir Crit Care Med 2018;198:1389–96. 10.1164/rccm.201803-0592oc 29889548

[27] Schultz SE, Rothwell DM, Chen Z et al Identifying cases of congestive heart failure from administrative data: a validation study using primary care patient records. Chronic Dis Inj Can 2013;33:160–6. 10.24095/hpcdp.33.3.06 23735455

[28] Howard M, Chalifoux M, Tanuseputro P. Does primary care model effect healthcare at the End of life? A population-based retrospective cohort study. J Palliat Med 2017;20:344–51. 10.1089/jpm.2016.0283 27893954

[29] Tanuseputro P, Beach S, Chalifoux M et al Associations between physician home visits for the dying and place of death: a population-based retrospective cohort study. PLoS One 2018;13:e0191322. 10.1371/journal.pone.0191322 29447291 PMC5813907

[30] Yarnell CJ, Fu L, Manuel D et al Association between immigrant Status and End-of-life care in Ontario, Canada. JAMA 2017;318:1479–88. 10.1001/jama.2017.14418 28973088 PMC5710367

[31] Weiner JP . The John Hopkins ACG® Case-Mix System Version 10.0 Release Notes.: The Johns Hopkins. Baltimore, MD: University Bloomberg School of Public Health, Health Services Research & Development Center, 2013.

[32] Weiner JP . The John Hopkins ACG® Case-Mix System Documentation & Application Manual Version 10.0. Baltimore, MD: The John Hopkins University Bloomberg School of Public Health, Health Services Research & Development Center, 2013.

[33] Singer AE, Goebel JR, Kim YS et al Populations and interventions for palliative and End-of-life care: a systematic review. J Palliat Med 2016;19:995–1008. 10.1089/jpm.2015.0367 27533892 PMC5011630

[34] Moorin RE, Youens D, Preen DB et al The association between general practitioner regularity of care and ‘high use’ hospitalisation. BMC Health Serv Res 2020;20:915. 10.1186/s12913-020-05718-0 33023571 PMC7541210

[35] Moorin RE, Youens D, Preen DB et al Association between continuity of provider-adjusted regularity of general practitioner contact and unplanned diabetes-related hospitalisation: a data linkage study in New South Wales, Australia, using the 45 and up study cohort. BMJ Open 2019;9:e027158. 10.1136/bmjopen-2018-027158 PMC6561442 31171551

