# Supplementary material for: Measuring the relationship between outpatient family physician visit regularity and acute care utilization during the end of life: a population-level retrospective cohort study
Source: Fam Pract. 2026 Apr 7;43(3):cmag014. doi: 10.1093/fampra/cmag014 (PMC13064537; doi:10.1093/fampra/cmag014)
Supplement: cmag014_Supplementary_Data [file cmag014_supplementary_data.zip › Appendices 2026-03-17.pdf]

Appendix 1: Description of health administrative data files held at ICES used in the study.

DATABASE	DATABASE CONTENT
**Ontario Health Insurance Plan database**	This dataset includes all claims by Ontario physicians for inpatient and ambulatory visits, consultations, and procedures. The data also include claims from optometrists for publicly funded reimbursement and from laboratories for all diagnostic tests performed.
**Ontario Registered Persons Database**	Demographic, place of residence and vital status information for all persons eligible to receive insured health services in the province, including date of birth, sex, and home address.
**Client Agency Program Enrolment Dataset**	This dataset details a list of patients registered to a primary care organization and identifies an association with a specific primary care physician and what type of primary care organization.
**Discharge Abstract Database**	This database contains administrative, clinical, and demographic information on all hospital discharges.
**National Ambulatory Care Reporting System**	This database contains administrative, clinical, and demographic information for all hospitals and community-based ambulatory care provision, including emergency departments.

Appendix 2: Relative variance index equation formula.

$$rvi = 1 \,/\, (1 + \frac{sd(Days)}{Mean(Days)} * 100)$$

where $Days$ is the number of days between encounters to a single physician.

Appendix 3: Multivariable associations between outpatient family physician visit relative variance index scores and acute care utilization during the last 30 days of life.

Variable	Hospitalizations, IRR (95% CI)	Emergency Department Visits, IRR (95% CI)	Acute Care Death, OR (95% CI)
**Quintiles of family physician visit RVI,**			
**Q1: 0.002 - 0.007 (ref)**			
**Q2: 0.008 - 0.009**	1.02 (1.00, 1.05)	1.02 (1.00, 1.04)	1.10 (1.06, 1.14)
**Q3: 0.010 - 0.011**	1.04 (1.01, 1.06)	1.04 (1.01, 1.06)	1.18 (1.14, 1.22)
**Q4: 0.012 - 0.015**	1.05 (1.03, 1.08)	1.05 (1.03, 1.08)	1.24 (1.19, 1.28)
**Q5: >0.015**	1.07 (1.05, 1.10)	1.06 (1.03, 1.08)	1.30 (1.26, 1.35)
**Age,**			
**19-44 (ref)**			
**45-54**	1.05 (0.96, 1.14)	0.98 (0.90, 1.06)	0.98 (0.85, 1.13)
**55-64**	1.09 (1.01, 1.18)	0.99 (0.92, 1.06)	0.97 (0.85, 1.10)
**65-74**	1.14 (1.06, 1.23)	1.03 (0.96, 1.11)	1.07 (0.94, 1.21)
**75-84**	1.16 (1.08, 1.25)	1.05 (0.98, 1.13)	1.04 (0.92, 1.18)
**85-94**	1.15 (1.07, 1.24)	1.06 (0.99, 1.14)	0.89 (0.78, 1.00)
**95+**	1.04 (0.95, 1.13)	0.97 (0.90, 1.05)	0.63 (0.55, 0.72)
**Male vs. female (ref)**	1.00 (0.98, 1.01)	1.01 (1.00, 1.03)	1.06 (1.04, 1.09)
**Neighbourhood income quintile status,**			
**Q1 – lowest (ref)**			
**Q2**	1.00 (0.98, 1.02)	0.99 (0.98, 1.01)	0.98 (0.95, 1.02)
**Q3**	0.99 (0.97, 1.01)	0.99 (0.97, 1.01)	0.94 (0.91, 0.97)
**Q4**	0.99 (0.97, 1.01)	0.98 (0.96, 1.00)	0.91 (0.87, 0.94)
**Q5 – highest**	0.97 (0.95, 0.99)	0.97 (0.95, 0.99)	0.87 (0.84, 0.91)
**Rural vs. urban (ref)**	1.05 (1.03, 1.07)	1.06 (1.04, 1.08)	1.20 (1.16, 1.25)
**Received provincial homecare services, yes vs. no (ref)**	0.79 (0.77, 0.80)	0.82 (0.80, 0.83)	0.48 (0.47, 0.50)
**Cancer prevalence, yes vs. no (ref)**	0.97 (0.95, 0.98)	0.97 (0.95, 0.98)	0.81 (0.79, 0.82)
**Quintiles of prevalent adjusted diagnostic groups,**			
**Q1: 1 - 9 (ref)**			
**Q2: 10 - 12**	1.56 (1.52, 1.60)	1.29 (1.26, 1.32)	2.14 (2.07, 2.22)
**Q3: 13 - 14**	1.75 (1.71, 1.80)	1.37 (1.34, 1.40)	3.07 (2.96, 3.19)
**Q4: 15 - 16**	1.83 (1.78, 1.88)	1.39 (1.36, 1.42)	3.85 (3.70, 4.01)
**Q5: >16**	1.88 (1.83, 1.93)	1.39 (1.35, 1.42)	4.83 (4.63, 5.03)
**Cardiorespiratory condition subgroups,**			
**ACOPD & HF (ref)**			
**ACOPD only**	0.91 (0.89, 0.94)	0.91 (0.89, 0.94)	0.65 (0.62, 0.68)

**HF only**	0.99 (0.97, 1.01)	0.96 (0.94, 0.98)	1.22 (1.19, 1.26)
**Quintiles of family physician visits,**			
**Q1: 2 - 4 (ref)**			
**Q2: 5 - 7**	1.01 (0.98, 1.03)	1.02 (1.00, 1.04)	1.00 (0.97, 1.04)
**Q3: 8 - 11**	1.01 (0.98, 1.03)	1.02 (1.00, 1.05)	0.93 (0.90, 0.97)
**Q4: 12 - 17**	0.99 (0.97, 1.01)	1.03 (1.01, 1.05)	0.87 (0.84, 0.91)
**Q5: >17**	0.95 (0.93, 0.98)	1.01 (0.99, 1.03)	0.75 (0.73, 0.78)
**Quintiles of specialist visits,**			
**Q1: 2 - 5 (ref)**			
**Q2: 6 - 9**	0.96 (0.94, 0.98)	0.97 (0.95, 0.99)	0.92 (0.89, 0.96)
**Q3: 10 - 15**	0.93 (0.91, 0.95)	0.94 (0.92, 0.96)	0.84 (0.81, 0.87)
**Q4: 16 - 24**	0.91 (0.89, 0.93)	0.93 (0.91, 0.95)	0.77 (0.74, 0.80)
**Q5: >24**	0.89 (0.86, 0.91)	0.90 (0.87, 0.92)	0.65 (0.62, 0.68)
**Akaike Information Criterion**	**245,700**	**253,200**	**173,300**

Notes: ACOPD = advanced chronic obstructive pulmonary disease; HF = heart failure; RVI = relative variance index; IRR = incidence rate ratio; OR = odds ratio; 95% CI = 95% confidence interval.

Appendix 4: Multivariable associations between outpatient family physician visit relative variance index scores (including adjusting for specialist visit regularity) and acute care utilization during the last 30 days of life.

Variable	Hospitalizations, IRR (95% CI)	Emergency Department Visits, IRR (95% CI)	Acute Care Death, OR (95% CI)
**Quintiles of family physician visit RVI,**			
**Q1: 0.002 - 0.007 (ref)**			
**Q2: 0.008 - 0.009**	1.01 (0.99, 1.03)	1.01 (0.99, 1.03)	1.08 (1.05, 1.12)
**Q3: 0.010 - 0.011**	1.02 (1.00, 1.05)	1.02 (1.00, 1.05)	1.14 (1.10, 1.19)
**Q4: 0.012 - 0.015**	1.03 (1.01, 1.06)	1.03 (1.01, 1.06)	1.18 (1.14, 1.23)
**Q5: >0.015**	1.03 (1.01, 1.06)	1.03 (1.01, 1.06)	1.23 (1.19, 1.28)
**Quintiles of specialist visit RVI,**			
**Q1: 0.001 - 0.006 (ref)**			
**Q2: 0.007 - 0.008**	1.10 (1.07, 1.12)	1.11 (1.09, 1.14)	1.30 (1.26, 1.35)
**Q3: 0.009 - 0.010**	1.15 (1.12, 1.17)	1.17 (1.15, 1.20)	1.46 (1.41, 1.51)
**Q4: 0.011 - 0.012**	1.19 (1.16, 1.21)	1.21 (1.18, 1.23)	1.62 (1.56, 1.68)
**Q5: >0.012**	1.25 (1.22, 1.28)	1.27 (1.24, 1.30)	1.82 (1.76, 1.89)
**Age,**			
**19-44 (ref)**			
**45-54**	1.04 (0.95, 1.14)	0.97 (0.90, 1.06)	0.96 (0.83, 1.11)
**55-64**	1.08 (1.00, 1.17)	0.98 (0.92, 1.06)	0.95 (0.84, 1.08)
**65-74**	1.13 (1.05, 1.22)	1.02 (0.95, 1.09)	1.04 (0.92, 1.17)
**75-84**	1.14 (1.06, 1.23)	1.03 (0.96, 1.11)	0.99 (0.87, 1.12)
**85-94**	1.13 (1.05, 1.22)	1.04 (0.97, 1.11)	0.83 (0.73, 0.94)
**95+**	1.02 (0.93, 1.11)	0.95 (0.88, 1.03)	0.59 (0.51, 0.67)
**Male vs. female (ref)**	1.00 (0.98, 1.01)	1.02 (1.00, 1.03)	1.06 (1.04, 1.09)
**Neighbourhood income quintile status,**			
**Q1 – lowest (ref)**			
**Q2**	1.00 (0.98, 1.02)	1.00 (0.98, 1.02)	0.99 (0.96, 1.02)
**Q3**	0.99 (0.97, 1.01)	0.99 (0.97, 1.01)	0.94 (0.91, 0.98)
**Q4**	0.99 (0.97, 1.01)	0.98 (0.96, 1.00)	0.91 (0.88, 0.94)
**Q5 – highest**	0.97 (0.95, 0.99)	0.97 (0.95, 0.99)	0.88 (0.85, 0.91)
**Rural vs. urban (ref)**	1.04 (1.02, 1.07)	1.05 (1.03, 1.08)	1.19 (1.15, 1.24)
**Received provincial homecare services, yes vs. no (ref)**	0.80 (0.78, 0.81)	0.83 (0.82, 0.84)	0.50 (0.49, 0.52)
**Cancer prevalence, yes vs. no (ref)**	0.96 (0.94, 0.97)	0.96 (0.94, 0.97)	0.78 (0.76, 0.80)
**Quintiles of prevalent adjusted diagnostic groups,**			
**Q1: 1 - 9 (ref)**			
**Q2: 10 - 12**	1.58 (1.54, 1.62)	1.30 (1.27, 1.34)	2.22 (2.14, 2.31)

**Q3: 13 - 14**	1.78 (1.73, 1.82)	1.39 (1.36, 1.42)	3.23 (3.10, 3.35)
**Q4: 15 - 16**	1.86 (1.81, 1.91)	1.41 (1.38, 1.45)	4.06 (3.91, 4.23)
**Q5: >16**	1.91 (1.86, 1.96)	1.41 (1.38, 1.45)	5.12 (4.91, 5.34)
**Cardiorespiratory condition subgroups,**			
**ACOPD & HF (ref)**			
**ACOPD only**	0.92 (0.90, 0.95)	0.93 (0.90, 0.95)	0.67 (0.64, 0.70)
**HF only**	0.99 (0.97, 1.01)	0.96 (0.95, 0.98)	1.24 (1.20, 1.28)
**Quintiles of family physician visits,**			
**Q1: 2 - 4 (ref)**			
**Q2: 5 – 7**	1.01 (0.98, 1.03)	1.02 (1.00, 1.04)	1.00 (0.96, 1.04)
**Q3: 8 – 11**	1.00 (0.98, 1.02)	1.02 (1.00, 1.04)	0.93 (0.89, 0.96)
**Q4: 12 – 17**	0.98 (0.96, 1.00)	1.02 (1.00, 1.05)	0.86 (0.83, 0.90)
**Q5: >17**	0.95 (0.93, 0.97)	1.01 (0.98, 1.03)	0.74 (0.71, 0.77)
**Quintiles of specialist visits,**			
**Q1: 2 - 5 (ref)**			
**Q2: 6 - 9**	0.96 (0.94, 0.98)	0.98 (0.96, 1.00)	0.93 (0.90, 0.97)
**Q3: 10 - 15**	0.93 (0.91, 0.95)	0.95 (0.93, 0.97)	0.85 (0.82, 0.88)
**Q4: 16 - 24**	0.91 (0.89, 0.94)	0.93 (0.91, 0.95)	0.78 (0.75, 0.81)
**Q5: >24**	0.89 (0.86, 0.91)	0.89 (0.87, 0.92)	0.64 (0.62, 0.67)
**Akaike Information Criterion**	**245,300**	**252,700**	**172,100**
Notes: ACOPD = advanced chronic obstructive pulmonary disease; HF = heart failure; RVI = relative variance index; IRR = incidence rate ratio; OR = odds ratio; 95% CI = 95% confidence interval.			

Appendix 5: Correlation between outpatient family physician visit relative variance index scores and outpatient care use during the last two years of life (truncating the last 30 days).

Variable	Family physician visit RVI	Family physician visits	Specialist visit RVI	Specialist visits	Unique specialists
**Family physician visit RVI**	1.000				
**Family physician visits**	-0.041	1.000			
**Specialist visit RVI**	0.020	-0.011	1.000		
**Specialist visits**	-0.039	0.113	-0.072	1.000	
**Unique specialists**	-0.048	0.134	-0.104	0.791	1.000
Note: RVI = relative variance index					

Appendix 6: Sensitivity analyses of the relative variance index scores using different time periods during the last two years of life.

Time Period	Family physician visit RVI	Specialist visit RVI
**Last two years of life (truncating the last 30 days), Median (P25, P75)**	0.011 (0.009, 0.014)	0.009 (0.007, 0.011)
**Last two years of life (truncating the last 90 days), Median (P25, P75)**	0.009 (0.007, 0.011)	0.006 (0.005, 0.008)
**Two years before death to one year before death, Median (P25, P75)**	0.003 (0.002, 0.004)	0.002 (0.002, 0.002)
**One year before death to 90 days before death, Median (P25, P75)**	0.007 (0.006, 0.009)	0.005 (0.004, 0.006)
**Last 90 days of life, Median (P25, P75)**	0.011 (0.008, 0.014)	0.008 (0.006, 0.010)

Notes: RVI = relative variance index; P25 = 25th percentile; P75 = 75th percentile.
